# Molecular Characterization and Prevalence of Antimicrobial-Resistant *Escherichia coli* Isolates Derived from Clinical Specimens and Environmental Habitats

**DOI:** 10.3390/microorganisms11061399

**Published:** 2023-05-26

**Authors:** Chrysoula Dioli, Olga Pappa, Eirini Siatravani, Spyridoula Bratakou, Apostolos Tatsiopoulos, Panagiota Giakkoupi, Vivi Miriagou, Apostolos Beloukas

**Affiliations:** 1 Molecular Microbiology and Immunology Laboratory, Department of Biomedical Sciences, University of West Attica, 28 Ag. Spyridonos Str., 12243 Athens, Greece; 2 Laboratory of Bacteriology, Hellenic Pasteur Institute, 127 Vasilissis Sofias Avenue, 11521 Athens, Greece; 3 Microbiology Laboratory, General Hospital of Livadeia, Agiou Vlasiou, 32100 Livadia, Greece; 4 Department of Public Health Policy, School of Public Health, University of West Attica, 196 Alexandras Avenue, 11521 Athens, Greece

**Keywords:** *E. coli*, environment, antibiotic resistance, antibiotic resistance genes

## Abstract

Antibiotic-resistant bacteria (ARB) are present in wastewaters as their elimination during treatment in wastewater treatment plants (WWTPs) is often impossible. Water plays an important role in the spread of these microorganisms among humans, animals and the environment. This study aimed to assess the antimicrobial resistance patterns, resistance genes and molecular genotypes by means of phylogenetic groups of *E. coli* isolates in aquatic habitats, including sewage and receiving water bodies, as well as clinical settings in the Boeotia regional district of Greece. The highest resistance rates among both environmental and clinical isolates were observed to be for penicillins, ampicillin and piperacillin. Resistance patterns related to extended spectrum β-lactamases (ESBL) production and ESBL genes were also detected in both environmental and clinical isolates. Phylogenetic group B2 was predominant in clinical settings and the second most frequent among wastewaters, whereas group A was dominant in all environmental isolates. In conclusion, the studied river water and wastewaters may serve as reservoirs of resistant *E. coli* isolates that pose potential threats to both human and animal health.

## 1. Introduction

The emergence of antimicrobial resistance (AR) to nearly all clinically relevant antibiotics is a pressing health risk that could reverse a century of medical progress. AR reduces the effectiveness of antimicrobials, leading to higher morbidity and mortality rates [1,2]. Antibiotic-resistant bacteria (ARB) are not confined to clinical settings, but are also spread via various routes in the ecosystem [3,4,5,6,7]. This is primarily triggered by the selective pressure of antimicrobial use in human and veterinary medicine, agriculture and aquaculture [8,9,10]. Significant amounts of antimicrobial residues are released into the environment via various routes, including discharges from wastewater treatment plants (WWTPs), disturbing the balance between sensitive and resistant bacteria populations [11,12,13,14].

WWTPs receive vast quantities of municipal and industrial waste daily, including hospital wastewater (HWW) that contains ARB and antibiotic resistance genes (ARGs) [1,14,15,16]. ARB and ARGs can evade treatment, and WWTP effluents (a) provide optimal conditions for ARB proliferation and the horizontal transfer of ARGs, (b) are often discharged into water bodies such as rivers, seas and lakes and (c) are reclaimed for industrial or irrigation purposes, in many countries, thus contributing to the further spread of AR in the ecosystem [3,17,18]. The systematic monitoring of wastewater provides valuable information regarding the presence and release of ARB into the environment, which is crucial for the safe reuse of treated wastewater [2,7]. Moreover, wastewater surveillance reveals the ARB and ARGs that are spreading in the community.

While the resistance of *E. coli* to last-line antibiotics that are widely used in clinical practice, livestock farming and aquaculture has been well studied in the hospital environment, information about community and environmental settings remains limited. *E. coli* can cause severe infections both in humans and animals, but is also a member of the autochthonous microbiota. *E. coli* also represents a major reservoir of resistance genes, which can lead to treatment failures in human medicine. A number of resistance genes have been identified in *E. coli*, and many of these genes can be horizontally transferred. Furthermore, *E. coli* can act as a donor and as a recipient of resistance genes. The transmission of virulent and resistant *E. coli* strains between aquatic environments and humans is a major concern, and can occur via various pathways such as direct contact or via the food chain. Thus, the circulation, phylogenies and dispersal of antibiotic-resistant *E. coli* isolates in environmental habitats are becoming increasingly important to be studied and monitored [3,4,6,19].

To our knowledge, the prevalence and diversity of ARGs in environmental *E. coli* isolates in Greece are still limited. Therefore, we aimed to assess AR patterns and detect the ARGs related to resistant phenotypes, identify molecular genotypes and compare resistance patterns and genotypes between clinical and environmental *E. coli* isolates from the Livadeia area (Greece). The study also aimed to detect common phenotypes and clones among the studied settings, both clinical and environmental, in Greece.

## 2. Materials and Methods

### 2.1. Sampling Locations and Collected Samples

**Figure 1:**
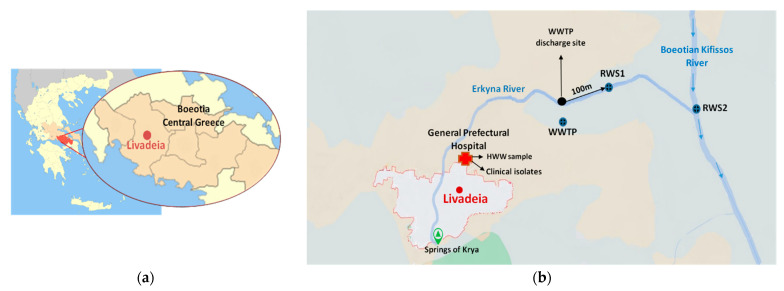
(**a**) The Boeotia regional unit is located in Central Greece and is divided into six municipalities. Livadeia serves as the capital of the Boeotia regional district. (**b**) The map on the right depicts the sampling locations and their relationships. The abbreviations used in the map are as follows: HWW, hospital wastewater; WWTP, wastewater treatment plant; RWS1, river water site 1 (located 100 m downstream from the WWTP discharge site); RWS2, river water site 2 (located 6 km downstream from the WWTP discharge site).

The sampling locations and collection procedures were carefully planned to capture the spread of environmental AR in Livadeia city (the capital of the regional unit) of the Boeotia regional district, Greece (Figure 1a). This region was chosen due to its intensive agricultural and farming activities, and it is crossed by two rivers: the Erkyna river on the northern side of the city and the Boeotian river on the southeast side of the city. The area also hosts a WWTP and a general prefectural hospital which performs a semi-treatment on the HWW. The hospital provides a wide range of services to approximately 60,000 people annually, including emergency and outpatient care, and has clinics for nephrology, pathology, cardiology, surgery, orthopedics and obstetrics–gynecology. After preliminary sedimentation, the hospital sewage is discharged into the regional WWTP.

The WWTP receives urban and HWW, with an average daily volume of 5500 m^3^/day at the entrance and an average hourly flow of 400 m^3^/h. It performs primary treatment, including screening, grit collection, grease trap, oxidation ditch and primary sedimentation, as well as biological treatment that includes nitrogen and phosphorus removal, secondary sedimentation, chlorination, sludge thickening and dewatering [15]. The secondary treated effluents are disposed of in the Erkyna river, and are used for the restricted irrigation of cropland during the irrigation season. The Erkyna river is directly influenced by the WWTP discharges and indirectly by the hospital sewage. The Erkyna river flows into the Boeotian Kifissos river at a point approximately 6 km away from the WWTP. Both rivers are used for irrigation purposes, with one irrigation project covering 16,000 acres of the studied area [20].

During the period of summer 2019 to spring 2021, six sequential sampling events were conducted in Livadeia city, Boeotia regional district. A total of four samples per sampling period were collected, including (a) semi-treated HWW from a septic tank outside the hospital, (b) wastewater at the outlet of the regional WWTP, (c) river water samples from the Erkyna river adjacent to the WWTP (RWS1) and (d) river water samples from the Boeotian Kifissos river at the junction with the Erkyna river (RWS2), located 6 km downstream from the WWTP (Figure 1b). A total of twelve river water samples (six from RWS1 and six from RWS2), six wastewater samples and six HWW samples were collected and analyzed. All of the samples were collected in sterile dark bottles (500 mL volume), were placed on ice and analyzed within 12 h post-collection. In addition, clinical isolates were collected from clinical specimens such as urine, blood and tissue from the microbiological laboratory of the hospital during the whole study period.

### 2.2. E. coli Isolation and Identification

*E. coli* isolation and identification were conducted using a standard membrane filtration technique (ISO 9308.01-1: 2017 [21]) for all river and wastewater samples. The procedure involved filtering multiple volumes (river water: 100 mL, 10 mL, 1 mL, wastewater: 10 mL, 1 mL, 0.1 mL) of each sample using a mixed cellulose ester membrane with a diameter of 47 mm and pore size of 0.45 μm (Whatman^®^ ME 25/21 ST). The membrane filters were then placed in Chromogenic Coliform medium (CHROMagar^TM^ CCA, EF342, Paris, France) with and without an antibiotic (CCA with 100 μg/mL ampicillin, CCA/AMP). In both culture media with and without AMP, all colonies showing positive β-d-galactosidase and β-d-glucuronidase reactions (dark blue to violet) were counted as *E. coli*. The CCA/AMP was used for the estimation and collection of the β-lactam-resistant isolates, while CCA without AMP was used for the enumeration and isolation of all *E. coli* isolates (e.g., sensitive and resistant to all antibiotics). The final confirmation of identification of all isolates was achieved using the indole biochemical test and molecular identification targeting the housekeeping β-d-glucuronidase gene *uidA* [22].

As for the clinical strains, they were obtained from biological fluids of hospitalized or emergency room patients, such as blood, urine and tissue, and were identified as *E. coli* in the microbiological laboratory of the hospital. Specifically, the clinical samples were cultivated on blood (Blood Agar Base CE, NCM2014, Neogen^®^, Lansing, MI, USA) and MacConkey agar (MacConkey Agar No. 3 CE, NCM2018, Neogen^®^) at 37 °C for 24 h. Following this, the isolates were identified via a Micro Scan automated system according to standard biochemical tests. The isolates were stored in cryovials with brain heart infusion (Scharlau Microbiology 02-599) +20% glycerol solution and transported to the Molecular Microbiology and Immunology Laboratory with proper packaging and transfer conditions [23].

### 2.3. Antibiotic Susceptibility Testing

All isolates (environmental and clinical) were tested for their antimicrobial susceptibility via disk diffusion assays (Kirby–Bauer method) in 18 antibiotics, commonly used in clinical practice, distributed in 9 different categories: penicillins (ampicillin (AMP; 10 μg), piperacillin (PIP; 30 μg)), penicillin/inhibitor combinations (amoxicillin/clavulanic acid (AMC; 20 μg/10 μg), piperacillin/tazobactam (TZP; 30 μg/6 μg)), cephalosporins (ceftriaxone (CRO; 30 μg), cefuroxime (CXM; 30 μg), ceftazidime (CAZ; 10 μg), cefotaxime (CTX; 5 μg), cefepime (FEP; 30 μg)), cephamycins (cefoxitin (FOX; 30 μg)), monobactams (aztreonam (ATM; 30 μg)), carbapenems (imipenem (IMP; 10 μg), meropenem (MEM; 10 μg)), aminoglycosides (amikacin (AN; 30 μg), gentamicin (GM; 10 μg)), quinolones (ciprofloxacin (CIP; 5 μg), nalidixic acid (NAL; 30 μg)) and miscellaneous agents (sulfamethoxazole-trimethoprim (SXT; 23.75 μg/1.25 μg)). The interpretation of the susceptibility results for the environmental and clinical isolates was performed according to EUCAST ECOFFs and clinical breakpoint criteria, respectively [24]. All isolates were characterized as sensitive/wild-type (S/WT: susceptible to all antibiotics), as non-wild-type (N-WT: resistant to only one antibiotic factor), as resistant (R: resistant to more than one antimicrobial agent; maximum of three different categories) or as multi-drug-resistant (MDR: resistant to at least one antimicrobial agent in more than three categories) [25,26]. ESBL production was detected phenotypically via a clavulanic acid synergy test (double-disk synergy test, DDST; synergy between amoxicillin/clavulanic acid (AMC) and ceftazidime (CAZ) or cefotaxime (CTX)) [27]. The phenotypic test and carbapenem inactivation method (CIM) were implemented in isolates which exhibited decreased susceptibility to carbapenems (meropenem, imipenem) in order to detect carbapenemase production such as KPC, NDM, OXA-48, VIM, IMP and OXA-23 [27,28].

### 2.4. Isolation of Genomic DNA

*E. coli* genomic DNA was extracted using either the boil–freezing method or the Purelink^ΤΜ^ Genomic DNA mini kit (Invitrogen, Waltham, MA, USA), following the manufacturer’s instructions, after 24 h of bacterial growth on nutrient agar.

### 2.5. PCR Amplification of Resistance Genes

All DDST-positive isolates underwent PCR to detect three different types of ESBL genes: *bla*_TEM_, *bla*_SHV_ and *bla*_CTX-M_ [29,30]. CIM-positive isolates were tested for the presence of carbapenemase genes (*bla*_KPC_, *bla*_VIM_, *bla*_NDM_, *bla*_IMP_, *bla*_OXA-48_ and *bla*_OXA-23_) [29,31]. Isolates resistant to penicillin/inhibitor combinations and cephamycins were tested for AmpC-type β-lactamases genes (*bla*_CMY_ and *bla*_FOX_) [27,32], while MDR isolates exhibiting resistance to SXT were screened for the dihydropteroate synthase gene (*sul1*) demonstrating resistance to sulphonamides [33]. PCR amplicons were subjected to Sanger sequence analysis (CeMIA SA, http://cemia.eu/sangersequencing.html, accessed on 12 September 2022), as previously described [26,34]. The sequences and chromatographs were interpreted using MEGA software (https://www.megasoftware.net/, accessed on 19 September 2022), and the BLAST algorithm (https://blast.ncbi.nlm.nih.gov/Blast.cgi, accessed on 18 September 2022) was used to identify antimicrobial resistance genes. DNA sequences were compared with reference antibiotic resistance genes from NCBI (https://www.ncbi.nlm.nih.gov/pathogens/refgene, accessed on 19 September 2022) and phylogenetic trees were constructed using the maximum likelihood method to investigate any possible correlations.

### 2.6. Molecular Typing

#### 2.6.1. Phylogrouping

The Triplex PCR phylogrouping method utilizes the detection of *chuA* and *yjaA* genes and the DNA fragment TSPE4.C2 to classify *E. coli* isolates into four phylogenetic groups, A, B1, B2 and D, as per Clermont’s schema [35]. This method was employed to investigate the correlation between the origin of the sample (clinical specimens, HWW, WWTP effluents, RWS1 and RWS2) and the phylogenetic groups, and to assess the possible association between groups and specific resistance profiles.

#### 2.6.2. Pulsed Field Gel Electrophoresis (PFGE)

Representative MDR isolates were subjected to typing via PFGE. In total, 51 *E. coli* isolates, which were characterized as being MDR, derived from different environments (6 clinical isolates, 17 from HWW, 13 from WWTP effluents, 8 from RSW1 and 7 from RSW2) and belonging to different phylogenetic groups, were subjected to genomic typing. PFGE was performed according to the pulse net protocol [36]. The isolates were cultured in nutrient broth overnight at 37 °C, and treated with lysozyme at 37 °C for 1 h and then with proteinase K at 56 °C for overnight incubation. After four washing steps, the DNA was digested using rare-cutting restriction endonuclease XbaI (Thermo Fisher Scientific (30 units/reaction)) at 37 °C overnight. The produced fragments of the digested genomic DNA were separated on 1% agarose gels using PFGE. Genomic profiles were visualized via staining with GelRed (Biosna) and compared visually according to Tenover et al. [37].

### 2.7. Statistical Analysis

Pearson’s chi-square test (or Fisher’s exact test in case the expected values of any of the cells were below 5) was performed to examine the relationship between the phylogenetic groups and origin of the sample, and additionally between the phylogenetic groups and resistance profiles. The SPSS v.29 package was used for statistical analysis.

## 3. Results

### 3.1. E. coli Collection

The total number of *E. coli* colonies was determined by counting the number of characteristic colonies on the membrane filter according to ISO 9308.01-1:2017. A total of 610 colonies presumptive of *E. coli* (identified by their blue-violet color in CCA) were initially collected. Out of the 610 colonies, 502 (171 from WWTP, 105 from semi-treated HWW, 163 from RWS1 and 63 from RWS2 samples) were finally confirmed as being *E. coli* using the gold standard procedures [ISO 9308.01-1:2017] and molecular *uidA* confirmatory test [22]. In more detail, of the 502 confirmed *Ε. coli* isolates, 296 (92 from WWTP, 73 from HWW, 91 from RWS1 and 40 from RWS2 samples) were collected from CCA culture media without AMP and 206 (79 from WWTP, 32 from HWW, 72 from RWS1 and 23 from RWS2 samples) were collected from CCA/AMP. Regarding the clinical collection, a total of 139 *E. coli* isolates were identified and confirmed, with 104 derived from urine, 30 from blood and 5 from patients’ tissue.

### 3.2. Antimicrobial Susceptibility Profiles and Assessment of Resistance Mechanisms

Considering that *E. coli* has no intrinsic resistance mechanisms, all of the isolates (environmental and clinical) were classified into specific sub-categories. Regarding the environmental isolates, 40.4% (203/502) were characterized as WT, 2.8% (14/502) were characterized as N-WT, 36.5% (183/502) were characterized as R and 20.3% (102/502) were characterized as MDR. Regarding the clinical isolates, 40% (56/139) were characterized as S, 46% (64/139) were characterized as R and 14% (19/139) were characterized as MDR. The data for the characterization of the resistance profiles of the environmental and clinical samples are summarized in Table 1.

**Figure 2:**
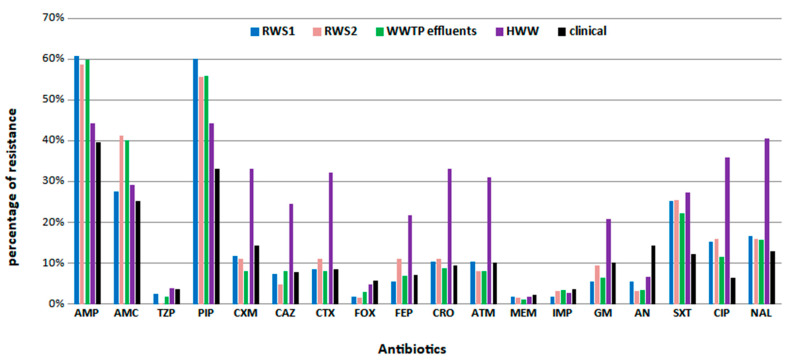
The frequency rate of resistance to each antibiotic per origin of sample. Abbreviations: AMP, ampicillin; AMC, amoxicillin/clavulanic acid; TZP, piperacillin/tazobactam; PIP, piperacillin; CXM, cefuroxime; CAZ, ceftazidime; CTX, cefotaxime; FOX, cefoxitin; FEP, cefepime; CRO, ceftriaxone; ATM, aztreonam; MEM, meropenem; IMP, imipenem; GM, gentamicin; AN, amikacin; SXT, sulfamethoxazole-trimethoprim; CIP, ciprofloxacin; NAL, nalidixic acid; HWW, hospital wastewater; WWTP, wastewater treatment plant; RWS1, river water site 1; RWS2, river water site 2.

The resistance frequencies of the 502 environmental and 139 clinical isolates in all of the tested antibiotics are presented in Figure 2. Resistance to penicillins (AMP and PIP) was the most frequent among all of the environmental and clinical isolates, followed by resistance to AMC. In more detail, 55% (275/502) of the environmental isolates exhibited resistance to AMP, 53% (267/502) exhibited resistance to PIP and 33% (164/502) exhibited resistance to AMC. A high resistance rate to quinolones (24.9%; 125/502) was also observed and the majority of the resistant isolates were derived from HWW (33.6%; 42/125) (Figure 2). Regarding the 139 clinical isolates, 40% (55/139) presented resistance to AMP, 33% (46/139) presented resistance to PIP and 25% (35/139) presented resistance to AMC. The number of different antibiotic categories in which environmental and clinical MDR isolates presented resistance is shown in Supplementary Table S1.

The resistance patterns exhibited by both environmental and clinical *E. coli* isolates were classified into two categories: multiple resistant patterns (MRPs; resistance patterns to more than three antibiotic categories) and resistant patterns (RPs; resistance patterns to maximum of three different antibiotic categories).

MRPs were further separated into six sub-categories: (a) MRP1—related to ESBL production, exhibiting resistance to penicillin/inhibitor combinations (such as AMC and TZP), expanded spectrum cephalosporins (such as CTX, CRO, CAZ and FEP) with or without resistance to monobactams (ATM) and positive DDST test; (b) MPR2—related to ESBL production, showing resistance to expanded spectrum cephalosporins (such as CTX, CRO, CAZ and FEP) with or without resistance to monobactams (ATM) and positive DDST test; (c) MRP3—related to ESBL+carbapenemase production, showing resistance to expanded spectrum cephalosporins, carbapenemes (IMP and ΜΕΜ) and positive DDST and CIM test; (d) MRP4—related to ESBL and AmpC production, showing resistance to cephamycins (FOX) and penicillin/inhibitor combinations (AMC and TZP) in addition to resistance to expanded spectrum cephalosporins; (e) MRP5—related to AmpC production, exhibiting resistance to cephamycins (FOX) and penicillin/inhibitor combinations (AMC and TZP) and negative DDST test and (f) other MRPs (MRP6–10) in which resistance to penicillins and to other non-β-lactam antibiotics (such as aminoglycosides, SXT and quinolones) was observed (Table 2). MRP2 and MRP1 were the most frequent MRPs among the MDR environmental and clinical isolates. Specifically, 32.3% (33/102) of the environmental MDR isolates presented an MRP2 pattern, while 29.4% (30/102) of the environmental and 36.8% (7/19) of the clinical MDR isolates presented an MRP1 pattern. Furthermore, fifty environmental and eight clinical isolates with ESBL-related MRPs presented concomitant resistance to quinolones (see Table 2).

Similarly, the RPs were further divided into five sub-categories: (a) RP1—related to ESBL production patterns with resistance to expanded spectrum cephalosporins and positive DDS test; (b) RP2—related to AmpC production with resistance to penicillins, penicillin/inhibitor combinations and cephamycin; (c) RPs3 (a–d), in which resistance to penicillins and to penicillin/inhibitor combinations with or without co-resistance to non-β-lactam antibiotics, such as quinolones, aminoglycosides and SXT, was observed; (d) RPs4 (a–g), in which resistance to penicillins with or without co-resistance to non-β-lactam antibiotics was observed and (e) RP5-6, in which only resistance to non-β-lactam antibiotics was observed (Supplementary Table S2). Our results show that RP3a was the most frequent RP among 183 R environmental (30.6%; 56/183) and among 64 R clinical isolates (29.6%; 19/64). Additionally, four R environmental isolates (two from RWS1 and two from RWS2) and one R clinical isolate were found to be potential ESBL producers.

### 3.3. Resistance Genes Detection

All of the β-lactamase producers (n = 80) were screened for β-lactamase genes. Thirty-two of the sixty-eight potential β-lactamase producers from the environment were isolated from HWW, while the remaining ones were derived from the WWTP effluents (n = 14), RWS1 (n = 15) and RWS2 (n = 7). Regarding the twelve clinical potential β-lactamase producers, eight, three and one were isolated from urine, blood and tissue, respectively. The characteristics of these isolates are shown in Supplementary Table S3. The *Bla*_CTX-M-group 1-type_ gene was detected in 52 isolates (65%; 52/80), the *bla*_CTX-M-group 9-type_ gene was identified in 7 isolates (9%, 7/80), the *Bla*_TEM_ gene was detected in 12 isolates (15%; 12/80) and the *bla*_SHV_ gene was detected in 17 isolates (21%; 17/80) (see Supplementary Table S3).

One isolate with an MRP3 profile was positive after the CIM test, indicating the presence of carbapenemase. Via molecular carbapenemase screening, the isolate was found to be positive for the *bla*_OXA-48-type_ gene, which was identified via sequencing coding for the OXA-244 enzyme (Supplementary Tables S3 and S4). In two isolates with MRP-4, the *bla*_CMY-2-type_ and *bla*_FOX-type_ genes were detected, coding for the AmpC-type enzymes, CMY-4 and FOX-17, respectively (Supplementary Tables S3 and S4). Detailed data for the detection rate of the β-lactamase genes in *E. coli* isolates derived from environmental and clinical samples are summarized in Table 3. The sequencing analysis confirmed the resistance genes with an identity value of 99% to 100% (Supplementary Table S4). Finally, the *sul1* gene was detected in 22/29 MDR isolates exhibiting resistance to SXT (5 clinical, 7 from HWW, 6 from WWTP, 3 from RWS1 and 1 from RWS2). 

### 3.4. Molecular Typing Analysis

**Figure 3:**
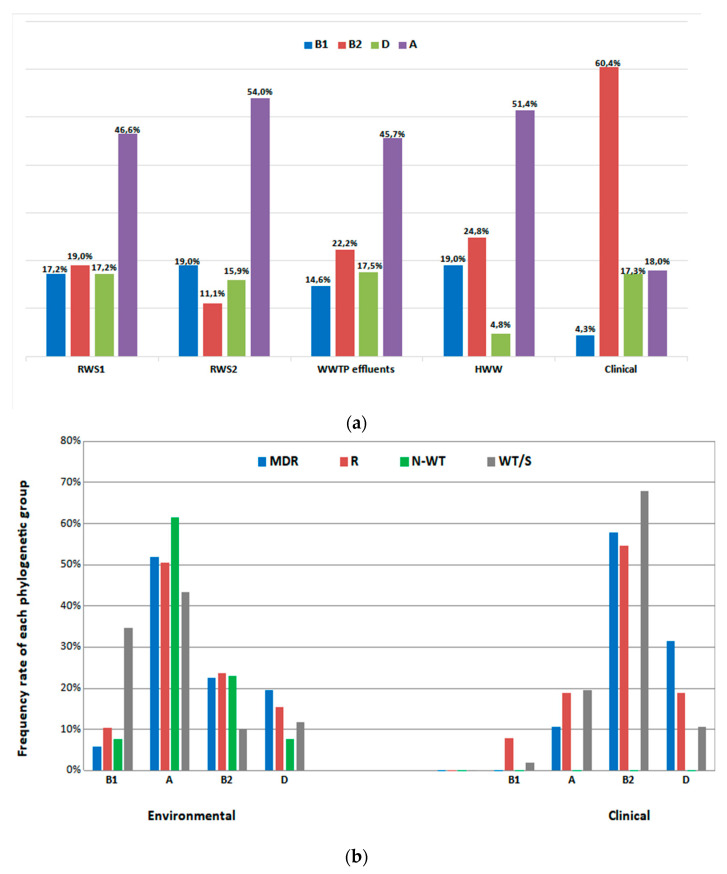
(**a**) The distribution of each phylogenetic group among different habitats (upper figure) and (**b**) the relationship between the phylogenetic groups and resistance profiles (lower figure). Abbreviations: RWS1, river water site 1; RWS2, river water site 2; WWTP, wastewater treatment plant; HWW, hospital wastewater; MDR, multi-drug-resistant; R, resistant; N-WT, non-wild-type; WT, wild-type.

There was a statistically significant correlation between the phylogenetic group and the origin of the sample [*X*^2^ (12, N = 641) = 110.63, *p* < 0.001)] (Supplementary Table S5a,b). Group A was the predominant group (48%, 242/502) in all of the environmental sample sources, followed by B2 (20%, 102/502), B1 (17%, 85/502) and D (15%, 73/502) (Figure 3a). Moreover, the occurrence of group B2 was higher in the *E. coli* isolates from wastewater samples (WWTP effluents and HWW) compared to other environmental sources, after evaluating the adjusted ratios (Supplementary Table S5a). In contrast to the environmental isolates, regarding the clinical isolates, group B2 was the predominant phylogenetic group (60%; 84/139), followed by A (18%, 25/139), D (17%, 24/139) and B1 (4%, 6/139) (Figure 3a). The above comparisons are in agreement with the adjusted ratios (Supplementary Table S5a).

A chi-square test of independence showed that there was an association between the phylogenetic group and the resistance profiles [*X*^2^ (18, N = 641) = 184.09, *p* < 0.001] (Supplementary Table S6a,b). Group A was the dominant group among all of the *E. coli* populations, including MDR, R, WT and N-WT, in environmental samples, while group B2 was dominant in the clinical isolates (among all of the populations, including MDR, R and S) (Supplementary Table S6a,b, Figure 3b). PFGE analysis revealed diverse genetic fingerprints (Supplementary Figure S1) and thus did not provide additional information on the molecular classification of the *E. coli* isolates.

For a number of isolates that produced the same β-lactamase and were derived from different sources, maximum likelihood phylogeny revealed the following: (a) *Bla*_CTX-M-1-like_ genes distributed the isolates into three groups, and all of the clinical isolates were clustered together into the same sub-group; (b) isolates which possessed the bla_CTX-M-9_ gene and were derived from RSW2 were grouped together and (c) isolates harboring the *bla*_SHV_ gene were divided into three clusters and the isolates from the RSW1 and WWTP effluents belonged to the same sub-group (see Supplementary Figure S2).

## 4. Discussion

R and MDR Enterobacterales pose an important human health issue due to the scarcity of available treatment options. In recent years, the One Health approach has been adopted to recognize the role of the environment in the dissemination of ARB, including ESBL-producing *E. coli* [2]. *E. coli* is a fundamental fecal indicator in monitoring the impact of effluents on the environment. Our analysis presents data that confirm that river water and reclaimed wastewater are reservoirs of R and MDR *E. coli* in commonly used antibiotics in clinical practice such as AMP, CIP, SXT and ESCs [8,18,19,38]. We also report antibiotic resistance to penicillins (AMP and PIP) as being the most frequent among both environmental and clinical isolates, although we also observed a high quinolone resistance rate in HWW [39,40,41].

ESBL-producing *E. coli*, specifically CTX-M-producing isolates (sub-types of the CTX-M-1 and CTX-M-9 groups), which are the predominant types in the studied habitats (environmental and clinical), are also widely found to be isolated from various aquatic environments (such as river and lakes) as well as hospitalized patients [39,40,42,43,44]. A portion of ESBL producers isolated from patients’ samples and wastewaters or river waters had the same resistance profiles, belonged to the same phylogenetic group and carried the same resistance gene (see Supplementary Table S3).

The phylogenetic group B2 has been previously reported to predominate in hospital environments [45,46,47]. Similarly, in our clinical isolates, the B2 phylogroup predominated and was also found to be the second most frequent group in HWW and WWTP effluents (Figure 3a, Supplementary Table S5a). The group B2 and group D isolates possessed the *chuA* gene [35], which is responsible for hemin utilization and has been identified in several pathogenic *E. coli* strains [48,49,50]. This fact implies a strong correlation between pathogenicity and phylogenetic groups B2 and D. In our study, a portion of clinical and environmental MDR and R isolates were classified into the phylogenetic groups B2 and D (Figure 3b), highlighting the high human health risks caused by exposing one to possible pathogenic R and MDR *E. coli* isolates derived from environmental sources such as rivers.

The reported results reveal that treated wastewater and river water are sources of resistant bacteria. The potential reuse of treated wastewater and river water exclusively for restricted crop irrigation, depending on the method of watering (e.g., spraying), may expose humans to the risk of developing gastroenteritis, particularly via droplet ingestion [51,52,53]. Regarding the risk of developing a urinary tract infection (UTI), *E. coli* is by far the most common cause for both community- and hospital-acquired UTIs. For UTI treatment, the recommended antimicrobials are SXT, CIP and AMC. In our study, *E. coli* strains that were found to be MDR, including those with co-resistance to SXT, CIP and AMC (Table 1), were not only isolated from the biological fluids of patients but also from all environmental habitats (see Figure 2). This fact demonstrates that human health risks can be caused by being exposed to MDR *E. coli* isolates present in waste and aquatic environments.

In our study, due to strict lockdown measures imposed during the COVID-19 pandemic, we were unable to carry out some samplings, which made seasonal analysis not feasible. Additionally, the molecular typing techniques employed did not provide adequate clustering information concerning the circulation of specific *E. coli* types between clinical settings and the environment. Nevertheless, this study represents the first systematic collection of *E. coli* isolates obtained from wastewater and river water samples from Livadeia, Greece, an area that combines urban life, husbandry and agriculture. Despite these limitations, this work provides valuable insights into the *E. coli* resistance profiles and genotypes present in wastewaters and aquatic habitats. The presence of AR *E. coli* isolates with the same MRPs in clinical and HWW samples sheds light on the spread of resistant bacteria in water bodies. The reported findings suggest a potential exchange of AR bacterial populations and similar AR determinants between clinical and environmental habitats. This raises concerns for public health, as aquatic environments could serve as reservoirs for the transmission of resistance genes to various bacterial species.

## Figures and Tables

**Table 1:** Resistance rate of environmental and clinical *E. coli* isolates.

Environmental Isolates	WT	N-WT	R	MDR
HWW (n = 105)	54.3% (57/105)	3.8% (4/105)	7.6% (8/105)	34.3% (36/105)
WWTP (n = 171)	36.8% (63/171)	3.5% (6/171)	43.3% (74/171)	16.4% (28/171)
RWS1 (n = 163)	36.8% (60/163)	1.2% (2/163)	45.4% (74/163)	16.6% (27/163)
RWS2 (n = 63)	36.5% (23/63)	3.1% (2/63)	42.9% (27/63)	17.5% (11/63)
**Clinical Isolates**	**S**		**R**	**MDR**
urine (n = 104)	41.3% (43/104)		45.2% (47/104)	13.5% (14/104)
blood (n = 30)	43.3% (13/30)		43.3% (13/30)	13.4% (4/30)
tissue (n = 5)	-		80% (4/5)	20% (1/5)

Abbreviations: WT, wild-type; N-WT, non-wild-type; S, sensitive; R, resistant; MDR, multi-drug-resistant; HWW, hospital wastewater; WWTP, wastewater treatment plant; RWS1, river water site 1; RWS2, river water site 2.

**Table 2:** Observed patterns of MDR isolates (MRPs, multiple resistant patterns).

			Environmental Isolates (Source)	Clinical Isolates
**MRP1**: **Related to ESBL production and resistance to penicillin/inhibitor combinations**		PEN/PEN–inhibitor/ESCs + SXT	1 (WWTP)	-
		PEN/PEN-inhibitor/ESCs + QNs	1 (HWW)	-
		PEN/PEN-inhibitor/ESCs/ATM	2 (1 RWS1, 1 RWS2)	-
		PEN/PEN-inhibitor/ESCs/ATM + QNs	9 (3 HWW, 3 WWTP, 3 RWS1)	-
		PEN/PEN-inhibitor/ESCs/ATM + AMG	2 (1 WWTP, 1 RWS2)	-
		PEN/PEN-inhibitor/ESCs/ATM + SXT	-	1
		PEN/PEN-inhibitor/ESCs/ATM + SXT + QNs	2 (1 HWW + 1 RWS2)	2
		PEN/PEN-inhibitor/ESCs/ATM + AMG + QNs	2 (HWW)	3
		PEN/PEN-inhibitor/ESCs/ATM + AMG + SXT + QNs	11 (HWW)	1
**Total MRP1: 37**			**30**	**7**
**MRP2**: Related to ESBL production		PEN/ESCs/ATM + QNs	8 (4 HWW, 3 RWS1, 1 RWS2)	-
		PEN/ESCs/ATM + AMG	3 (2 RWS1, 1 RWS2)	-
		PEN/ESCs/ATM + SXT	7 (6 WWTP, 1 RWS1)	-
		PEN/ESCs/ATM + SXT + QNs	2 (WWTP)	1
		PEN/ESCs/ATM + AMG + SXT + QNs	8 (6 HWW, 2 RWS1)	-
		PEN/ESCs/ATM + AMG + QNs	4 (HWW)	-
		PEN/ESCs + SXT + QNs	1 (WWTP)	-
**Total MRP2: 34**			**33**	**1**
**MRP 3**: Related to ESBL + carbapenemase production		PEN/PEN-inhibitor/ESCs/CARB/ATM + SXT	1 (RWS1)	-
**Total MRP3: 1**			**1**	**-**
**MRP 4**: Related to ESBL + AmpC β-lactamases production		PEN/PEN-inhibitor/ESCs/FOX/ATM	2 (1 HWW, 1 RWS1)	1
		PEN/PEN-inhibitor/ESCs/FOX/ATM + AMG + SXT	-	1
		PEN/PEN-inhibitor/ESCs/FOX/ATM + SXT + QNs	-	1
		PEN/PEN-inhibitor/ESCs/FOX + AMG + SXT + QNs	2 (HWW)	-
**Total MRP 4: 7**			**4**	**3**
**MRP 5**: Related to AmpC β-lactamases production		PEN/PEN-inhibitor/FOX + AMG + QNs	1 (WWTP)	-
		PEN/PEN-inhibitor/FOX + QNs	1 (WWTP)	-
		PEN/PEN-inhibitor/NSCs/FOX + AMG	2 (RWS1)	2
**Total MRP5: 6**			**4**	**2**
**MPR 6**	Susceptibility to cephalosporins Penicillinase production with resistance to other non-β-lactam antibiotics	PEN/PEN-inhibitor + SXT + QNs	9 (2 WWTP, 4 RWS1, 3 RWS2)	1
		**Total MRP6: 10**	9	1
**MRP 7**		PEN/PEN-inhibitor + AMG + SXT	10 (6 WWTP, 4 RWS1)	5
		**Total MRP7: 13**	**10**	**3**
**MRP 8**		PEN/PEN-inhibitor + AMG + SXT + QNs	7 (1 HWW, 1 WWTP, 2 RWS1, 3 RWS2)	2
		**Total MRP8: 9**	**7**	**2**
**MRP 9**		PEN/PEN-inhibitor + AMG + QNs	1 (WWTP)	-
		**Total MRP9: 1**	**1**	-
**MRP10**		PEN + AMG + SXT + QNs	3 (2 WWTP, 1 RWS1)	-
		**Total MRP10: 3**	**3**	-

Total MRPs: 121. Total environmental isolates with MRPs: 102, and total clinical isolates with MRPs: 19. Abbreviations: MRPs, multiple resistant patterns; ESBL, extended-spectrum-β-lactamase; PEN, penicillins; PEN–inhibitor, penicillin–inhibitor combinations; ESCs, extended spectrum cephalosporins; SXT, sulfamethoxazole-trimethoprim; QNs, quinolones; ATM, aztreonam; AMG, aminoglycosides; CARB, carbapenems; FOX, cefoxitin; NSCs, narrow spectrum cephalosporins; HWW, hospital wastewater; WWTP, wastewater treatment plant; RWS1, river water site 1; RWS2, river water site 2.

**Table 3:** Detection rate of β-lactamase genes among clinical and environmental isolates.

β-Lactamase Genes		Clinical Isolates	Environmental Isolates				Total
			HWW	WWTP Effluents	RWS1	RWS2	
**ESBL genes**	*bla*_CTX-M-group-1-type_	10	22	10	8	2	52
	*bla*_CTX-M-group-9-type_			1	3	3	7
	*bla*_SHV_	1	12	2	3		17
	*bla*_TEM_	4	3	2	3		12
**Carbapenemase genes**	*bla*_OXA-48-type_				1		1
**AmpC-type genes**	*bla*_CMY-2-type_		1				1
	*bla*_FOX-type_		1				1

## Data Availability

All data relevant to this work are available upon reasonable request.

## Supplementary Materials

The following supporting information can be downloaded at https://www.mdpi.com/article/10.3390/microorganisms11061399/s1: Table S1: The *E. coli* isolates from environmental habitats and clinical specimens that exhibit multi-drug resistance; Table S2: Observed patterns of R isolates (RPs, resistant patterns); Table S3: Characteristics of environmental and clinical isolates harboring β-lactamasegenes; Table S4: Sequencing results for the possible β-lactamase producers; Table S5a: Examination of the relationship between phylogenetic groups and origin of the sample; Sample and group crosstabulation; Table S5b: Examination of the relationship between phylogenetic groups and origin of the sample; Pearson’s chi-square test results; Table S6a: Examination of the relationship between phylogenetic groups and resistance profile; Resistance profile and group crosstabulation; Table S6b: Examination of the relationship between phylogenetic groups and resistance profiles; Pearson’s chi-square test results. Figure S1: PFGE analysis; Diverse PFGE patterns of *E. coli* isolated from clinical and environmental samples; Figure S2: Maximum likelihood phylogenetic trees for (A) *bla*_CTX-M-groups_, (B) *bla*_TEM_ and (C) *bla*_SHV_ nucleotide sequences.
