# Supplementary material for: Molecular Characterization and Prevalence of Antimicrobial-Resistant Escherichia coli Isolates Derived from Clinical Specimens and Environmental Habitats
Source: Microorganisms. 2023 May 26;11(6):1399. doi: 10.3390/microorganisms11061399 (PMC10305429; doi:10.3390/microorganisms11061399)
Supplement: Supplementary file 1 [file microorganisms-11-01399-s001.zip › microorganisms-2345541-supplementary.pdf]

microorganisms

Research Article

Molecular Characterization and Prevalence of Antimicrobial Resistant *Escherichia coli* Isolates Derived from Clinical Specimens and Environmental Habitats

Chrysoula Dioli[1], Olga Pappa[1], Eirini Siatravani[1, 2], Spyridoula Bratakou[1], Apostolos Tatsiopoulos[3], Panagiota Giakkoupi[4], Vivi Miriagou[2], Apostolos Beloukas[1,*]

[1] Molecular Microbiology and Immunology laboratory, Department of Biomedical Sciences, University of West Attica, 28 Ag. Spyridonos str 12243 Athens, Attica, Greece

[2] Hellenic Pasteur Institute, Laboratory of Bacteriology, 127 Vasilissis Sofias Avenue 11521, Athens, Greece

[3] Microbiology Laboratory, General Hospital of Livadeia, Agiou Vlasiou, 32100, Livadia, Viotia, Greece

[4] Department of Public Health Policy, School of Public Health University of West Attica, 196 Alexandras Avenue, 11521 Athens, Greece

* **Correspondence:** abeloukas@uniwa.gr

SUPPLEMENTARY MATERIAL

Supplementary Table S1: The *E. coli* isolates from environmental habitats and clinical specimens that exhibit multidrug resistance.
Supplementary Table S2: Observed patterns of R isolates (RP, resistant patterns).
Supplementary Table S3: Characteristics of environmental and clinical isolates harboring β-lactamase genes.
Supplementary Table S4: Sequencing results for the possible β-lactamase producers.
Supplementary Table S5a: Examination of the relationship between phylogenetic groups and origin of the sample; Sample and Group Crosstabulation.
Supplementary Table S5b: Examination of the relationship between phylogenetic groups and origin of the sample; Pearson's chi-square test results.
Supplementary Table S6a: Examination of the relationship between phylogenetic groups and resistance profile; Resistance profile and Group Crosstabulation.
Supplementary Table S6b: Examination of the relationship between phylogenetic groups and resistance profile; Pearson's chi-square test results.
Supplementary Figure S1: PFGE analysis; Diverse PFGE patterns of *E. coli* isolated from clinical and environmental samples.
Supplementary Figure S2: Maximum Likelihood phylogenetic trees for A) $bla_{CTX\text{-}M\text{-}groups}$, B) bla_{TEM} and C) bla_{SHV} nucleotide sequences

Supplementary Table S1. The *E. coli* isolates from environmental habitats and clinical specimens that exhibit multidrug resistance

Number of different antibiotic categories in which environmental MDR isolates presented resistance	Number of environmental isolates obtained from each sample source				
	HWW	WWTP effluents	RWS1	RWS2	Total
4 antibiotic categories	5	8	16	6	35
5 antibiotic categories	8	20	8	4	40
6 antibiotic categories	10		3		13
7 antibiotic categories	13			1	14
**Total MDR**	36	28	27	11	102

Number of different antibiotic categories in which clinical MDR isolates presented resistance	Number of clinical isolates per specimen type			
	Urine	Blood	Tissue	Total
4 antibiotic categories	2	1		3
5 antibiotic categories	5	1		6
6 antibiotic categories	5	1	1	7
7 antibiotic categories	2	1		3
**Total MDR**	14	4	1	19

Abbreviations: MDR, Multi-drug resistant; HWW, hospital wastewater; WWTP, wastewater treatment plant; RWS1, river water site 1; RWS2, river water site 2.

Supplementary Table S2: Observed patterns of R isolates (RP, resistant patterns)

			Environmental isolates (source)	Clinical Isolates	Total
**RP1**: Related to ESBL production		PEN/ ESCs	4 (2 RWS1, 2 RWS2)	-	4
		PEN/ ESCs/ ATM	-	2	2
**RP2**: Related to Ampc β-lactamases production		PEN/ PEN.-inhibitor/ FOX	-	1	1
**RP3a**		PEN/ PEN-inhibitor	56 (30 WWTP, 18 RWS1, 8 RWS2)	19	75
**RP3b**		PEN/ PEN-inhibitor + SXT	20 (3 HWW, 8 WWTP, 5 RWS1, 4 RWS2)	1	21
**RP3c**		PEN/ PEN-inhibitor + AMG	4 (3 WWTP, 1 RWS1)	2	6
**RP3d**		PEN/ PEN-inhibitor + QNs	18 (2 HWW, 9 WWTP, 3 RWS1, 4 RWS2)	-	18
**RP4a**		PEN	33 (13 WWTP, 18 RWS1, 2 RWS2)	8	41
**RP4b**		PEN + SXT	14 (2 WWTP, 10 RWS1, 2 RWS2)	4	18
**RP4c**		PEN + SXT+ QNs	11 (3 WWTP, 8 RWS1)	-	11
**RP4d**		PEN + QNs	10 (3 WWTP, 5 RWS1, 2 RWS2)	-	10
**RP4e**		PEN + AMG + QNs	-	1	1
**RP4f**		PEN + AMG	5 (1 WWTP, 2 RWS1, 2 RWS2)	1	5
**RP4g**		PEN + SXT + AMG	-	3	3
**RP5**	Resistant only to non β-lactam antibiotics	QNs	5 (3 HWW, 2 WWTP)	15	20
**RP6**		SXT	-	4	4
**RP6a**		SXT + QNs	3 (2 RWS1, 1 RWS2)	3	7

Abbreviations: RP, resistant patterns; ESBL, extended-spectrum-β-lactamase; PEN, penicillins; ESCs, extended-spectrum cephalosporins; ATM, aztreonam; PEN.-inhibitor, penicillin-inhibitor combination; FOX, cefoxitin; SXT, sulfamethoxazole-trimethoprim; AMG, aminoglycosides; QNs, quinolones; HWW, hospital wastewater; WWTP, wastewater treatment plant; RWS1, river water site 1; RWS2, river water site 2.

Supplementary Table S3. Characteristics of environmental and clinical isolates harboring β-lactamse genes

Isolate ID	Type of Sample/ Sampling site	Phylogenetic group	Resistance Pattern	Resistance Profile	DDST	β-lactamases
297	Treated Wastewater/ WWTP outlet	A	AMP, AMC, PIP, CAZ, CRO, ATM, AN	MDR	+	SHV
326	Treated Wastewater/ WWTP outlet	B2	AMP, PIP, MEM, IMP	R		TEM
344	HWW/ septic tank	A	AMP, PIP, CXM, CAZ, CTX, FEP, CRO, ATM, CIP, NAL	MDR	+	SHV
345	HWW/ septic tank	A	AMP, PIP, CAZ, CTX, CRO, ATM, NAL	MDR	+	SHV
367	River water/ RWS1	A	AMP, AMC, TZP, PIP, CXM, CAZ, CTX, FEP, FOX, CRO, ATM, MEM, IMP	MDR	-	-
405	River water/ RWS1	D	AMP, AMC, PIP, CXM, CTX, CRO, ATM, CIP, NAL	MDR	+	CTX-M group 1
408	River water/ RWS1	D	AMP, AMC, TZP, PIP, TCC, CXM, CAZ, CTX, FEP, CRO, ATM, MEM, SXT	MDR	+	CTX-M group 1, OXA-48
426	HWW/ septic tank	D	AMP, AMC, PIP, CXM, CAZ, CTX, CRO, ATM, CIP, NAL	MDR	+	SHV
427	HWW/ septic tank	B2	AMP, AMC, PIP, CXM, CAZ, CTX, CRO, ATM, AN, CIP, NAL	MDR	+	SHV
431	HWW/ septic tank	B2	AMP, PIP, CXM, CAZ, CTX, CRO, ATM, AN, CIP, NAL	MDR	+	SHV
434	HWW/ septic tank	B2	AMP, AMC, PIP, CAZ, CTX, CRO, ATM, CIP, NAL	MDR	+	SHV
436	HWW/ septic tank	B2	AMP, AMC, PIP, CXM, CAZ, CTX, CRO, ATM, AN, CIP, NAL	MDR	+	SHV
472	River water/ RWS1	A	AMP, AMC, PIP, CXM, CTX, CRO, ATM, NAL	MDR	+	CTX-M group 9, TEM
477	River water/ RWS1	B1	AMP, PIP, CXM, CAZ, CRO, ATM, CIP, NAL	MDR	+	SHV
491	Treated Wastewater/ WWTP outlet	A	AMP, AMC, PIP, CXM, CTX, CRO, FEP, ATM, NAL	MDR	+	CTX-M group 1
494	Treated Wastewater/	B1	AMP, AMC, PIP, CXM, CTX, CRO, SXT	MDR	+	TEM

	WWTP outlet					
497	Treated Wastewater/ WWTP outlet	A	AMP, AMC, PIP, CXM, CAZ, CTX, FEP, CRO, ATM, NAL	MDR	+	CTX-M group 1
506	Treated Wastewater/ WWTP outlet	D	AMP, PIP, CXM, CAZ, CTX, FEP, ATM, SXT, NAL	MDR	+	CTX-M group 1
510	Treated Wastewater/ WWTP outlet	B2	AMP, PIP, CXM, CTX, CRO, SXT, CIP, NAL	MDR	+	-
546	HWW/ septic tank	B2	AMP, AMC, PIP, CXM, CAZ, CTX, CRO, ATM, NAL	MDR	+	SHV
550	HWW/ septic tank	A	AMP, AMC, TZP, PIP, CXM, FOX, FEP, GM, SXT, CIP, NAL	MDR	-	-
553	HWW/ septic tank	A	AMP, AMC, PIP, CXM, CTX, CRO, ATM, SXT, CIP, NAL	MDR	+	CTX-M group 1
555	HWW/ septic tank	A	AMP, AMC, PIP, CXM, CAZ, CTX, FEP, CRO, ATM, GM, SXT, CIP, NAL	MDR	+	CTX-M group 1
556	HWW/ septic tank	A	AMP, PIP, CXM, CAZ, CTX, FEP, CRO, ATM, CIP, NAL	MDR	+	SHV
557	HWW/ septic tank	A	AMP, AMC, PIP, CXM, CTX, FEP, CRO, ATM, GN, SXT, CIP, NAL	MDR	+	CTX-M group 1, TEM, SHV
581	HWW/ septic tank	A	AMP, AMC, TZP, PIP, CXM, FOX, FEP, GM, SXT, NAL, CIP	MDR	-	FOX
594	River water/ RWS1	B1	AMP, PIP, CXM, CAZ, CTX, CRO, ATM, CIP, NAL	MDR	+	SHV
595	River water/ RWS1	B2	AMP, PIP, CXM, CAZ, CTX, FEP, CRO, ATM	MDR	+	CTX-M group 1
598	River water/ RWS1	D	AMP, PIP, CXM, CAZ, CTX, FEP, CRO, ATM, GN, SXT, NAL	MDR	+	CTX-M group 1
601	River water/ RWS1	A	AMP, PIP, CXM, CAZ, CTX, FEP, CRO	R	+	CTX-M group 9
602	River water/ RWS1	A	AMP, PIP, CXM, CAZ, CRO, ATM	MDR	+	-
610	River water/ RWS1	D	AMP, PIP, CXM, CAZ, CTX, FEP, CRO, ATM, GN, SXT, NAL	MDR	+	CTX-M group 1
611	River water/ RWS1	A	AMP, PIP, CXM, CTX, FEP, CRO	R	+	CTX-M group 9
614	River water/ RWS2	A	AMP, PIP, CXM, CTX, FEP, CRO	R	+	CTX-M group 9

616	River water/ RWS2	A	AMP, PIP, CXM, CTX, FEP, CRO	R	+	CTX-M group 9
618	River water/ RWS2	A	AMP, AMC, PIP, CXM, CAZ, CTX, FEP, CRO, ATM	MDR	+	CTX-M group 9
620	River water/ RWS2	A	AMP, PIP, CXM, CTX, FEP, CRO, ATM, AN	MDR	+	-
630	River water/ RWS2	A	AMP, AMC, PIP, CXM, CTX, FEP, CRO, ATM, AN	MDR	+	-
638	Treated Wastewater/ WWTP outlet	D	AMP, PIP, CXM, CAZ, CTX, FEP, CRO, ATM, SXT, NAL	MDR	+	CTX-M group 1
645	Treated Wastewater/ WWTP outlet	D	AMP, AMC, PIP, CXM, CAZ, CTX, FEP, CRO, ATM, NAL	MDR	+	CTX-M group 1
673	HWW/ septic tank	A	AMP, PIP, CAZ, CRO, ATM, NAL	MDR	+	SHV
678	HWW/ septic tank	A	AMP, AMC, PIP, CXM, CAZ, CTX, FEP, CRO, ATM, GN, SXT, CIP, NAL	MDR	+	CTX-M group 1
681	HWW/ septic tank	A	AMP, PIP, CXM, CTX, FEP, CRO, ATM, GN, SXT, CIP, NAL	MDR	+	CTX-M group 1
682	HWW/ septic tank	A	AMP, AMC, PIP, CXM, CAZ, CTX, FEP, CRO, ATM, GN, SXT, CIP, NAL	MDR	+	CTX-M group 1
683	HWW/ septic tank	A	AMP, AMC, PIP, CXM, CAZ, CTX, FEP, CRO, ATM, GN, SXT, CIP, NAL	MDR	+	CTX-M group 1
738	River water/ RWS1	B2	AMP, AMC, TZP, PIP, CXM, FOX, CAZ, GM, AN	MDR	-	TEM
739	River water/ RWS1	A	AMP, PIP, CXM, CAZ, CTX, CRO, ATM, CIP, NAL	MDR	+	SHV
740	River water/ RWS1	A	AMP, PIP, CXM, CAZ, CTX, FEP, CRO, ATM, SXT	MDR	+	CTX-M group 1
748	River water/ RWS1	D	AMP, AMC, PIP, CXM, CAZ, CTX, FEP, CRO, ATM, NAL	MDR	+	CTX-M group 1
785	River water/ RWS2	D	AMP, AMC, PIP, CXM, CAZ, CTX, FEP, CRO, ATM, IMP, SXT, NAL	MDR	+	CTX-M group 1
791	River water/ RWS2	D	AMP, PIP, CXM, CAZ, CTX, FEP, CRO, ATM, CIP, NAL	MDR	+	CTX-M group 1
792	Treated Wastewater/ WWTP outlet	A	AMP, PIP, CXM, CAZ, CTX, FEP, CRO, ATM, SXT	MDR	+	CTX-M group 1
801	Treated Wastewater/	A	AMP, PIP, CXM, CAZ, CTX, FEP, CRO, ATM,	MDR	+	CTX-M group 1

	WWTP outlet		SXT			
802	Treated Wastewater/ WWTP outlet	A	AMP, PIP, CXM, CAZ, CTX, FEP, CRO, ATM, SXT	MDR	+	CTX-M group 1
803	Treated Wastewater/ WWTP outlet	A	AMP, PIP, CXM, CAZ, CTX, FEP, CRO, ATM, SXT	MDR	+	CTX-M group 1
806	Treated Wastewater/ WWTP outlet	A	AMP, PIP, CXM, CAZ, CTX, FEP, CRO, ATM, STX	MDR	+	CTX-M group 1
810	Treated Wastewater/ WWTP outlet	B2	AMP, PIP, CXM, CAZ, CTX, FEP, CRO, ATM, STX	MDR	+	CTX-M group 9
838	HWW/ septic tank	A	AMP, PIP, CXM, CAZ, CTX, FEP, CRO, ATM, GM, SXT, CIP, NAL	MDR	+	CTX-M group 1
839	HWW/ septic tank	A	AMP, PIP, CXM, CAZ, CTX, FEP, CRO, ATM, GM, SXT, CIP, NAL	MDR	+	CTX-M group 1
852	HWW/s eptic tank	B2	AMP, PIP, CXM, CAZ, CTX, FEP, CRO, ATM, AN, CIP, NAL	MDR	+	CTX-M group 1
858	HWW/ septic tank	A	AMP, AMC, PIP, CXM, CAZ, CTX, FOX, CRO, ATM, IMP	MDR	-	CMY
117cli	Clinical/ urine	A	AMP, AMC, PIP, CXM, CAZ, CTX, FEP, CRO, ATM, STX	MDR	+	CTX-M group 1, TEM
203cli	Clinical/ blood	D	AMP, AMC, PIP CXM, CAZ, CTX, FOX, CRO, ATM, STX, NAL	MDR	+	CTX-M group 1, TEM
22cli	Clinical/ urine	B2	AMP, AMC, PIP, CXM, CAZ, CTX, FEP, CRO, ATM, AN (I), CIP, NAL	MDR	+	CTX-M group 1
294cli	Clinical/ urine	B2	AMP, PIP, CXM, CTX, CRO, ATM	R	+	CTX-M group 1
325cli	Clinical/ blood	A	AMP, PIP, CXM, CAZ, CTX, CRO, ATM	R	+	CTX-M group 1
333cli	Clinical/ wound	B2	AMP, AMC, PIP, CXM, CTX, FEP, CRO, ATM, GM, CIP, NAL	MDR	+	CTX-M group 1, SHV
350cli	Clinical/ urine	B2	AMP, PIP, CXM, CAZ, CTX, CRO, FEP, ATM, SXT, CIP, NAL	MDR	+	CTX-M group 1
54cli	Clinical/ urine	B2	AMP, AMC, PIP, TZP, TCC, CXM, CAZ, CTX, FEP, CRO, ATM, GM, AN, CIP, NAL	MDR	+	CTX-M group 1, TEM
59cli	Clinical/ blood	D	AMP, AMC, PIP, TZP, TCC, CXM, CAZ, CTX,	MDR	+	CTX-M-group 1, TEM

			FEP, CRO, ATM, SXT, NAL			
60cli	Clinical/ urine	B2	AMP, AMC, PIP, TCC, CXM, CTX (I), CRO, ATM (I), AN, SXT, CIP, NAL	MDR	+	CTX-M group 1
84cli	Clinical/ urine	D	AMP, AMC, PIP, CXM, CAZ, CTX, FEP, CRO, FOX, ATM	MDR	+	

Abbreviations: DDST, double disk synergy test; HWW, hospital wastewater; WWTP, wastewater treatment plant; RWS1, river water site 1; RWS2, river water site 2; AMP, ampicillin; AMC, amoxicillin/clavulanic acid; TZP, piperacillin/ tazobactam; PIP, piperacillin; CXM, cefuroxime; CAZ, ceftazidime; CTX, cefotaxime; FOX, cefoxitin; FEP, cefepime; CRO, ceftriaxone; ATM, aztreonam; MEM, meropenem; IMP, imipenem; GM, gentamicin; AN, amikacin; SXT, sulfamethoxazole-trimethoprim; CIP, ciprofloxacin; NAL, nalidixic acid; MDR, multi-drug resistant; R, resistant.

Supplementary Table S4. Sequencing results for the possible β-lactamase producers

E. coli isolates	NCBI Reference Sequence	*bla* gene	Query cover	Identity value
E. coli-22_CTX-M-group 1 (clinical/ urine)	NG_048935.1	$bla_{\text{CTX-M-15-like}}$ (ESBL)	99%	99.04%
E. coli-59_CTX-M-group 1 (clinical/ blood)	NG_048935.1	$bla_{\text{CTX-M-15-like}}$ (ESBL)	97%	99.27%
E. coli-60_CTX-M-group 1 (clinical/ urine)	NG_048935.1	$bla_{\text{CTX-M-15-like}}$ (ESBL)	99%	99.74%
E. coli-491_CTX-M group 1 (environmental/ WWTP)	NG_048897.1	$bla_{\text{CTX-M-1-like}}$ (ESBL)	96%	99.27%
E. coli-678_CTX-M-group 1 (environmental/ HWW)	NG_048935.1	$bla_{\text{CTX-M-15-like}}$ (ESBL)	95%	99.51%
E. coli-472_CTX-M-group 9 (environmental/ RWS1)	NG_049043.1	$bla_{\text{CTX-M-9-like}}$ (ESBL)	90%	99.39%
E. coli-616_CTX-M-group 9 (environmental/ RSW2)	NG_049043.1	$bla_{\text{CTX-M-9}}$ (ESBL)	90%	100%
E. coli-618_CTX-M-group 9 (environmental/ RSW2)	NG_049043.1	$bla_{\text{CTX-M-9}}$ (ESBL)	98%	100%
E. *coli*-858_CMY-2 like AmpC (environmental/ HWW)	NG_048834.1	$bla_{\text{CMY-4-like}}$ (AmpC type β-lactamases)	97%	99.81%
E. coli-581_FOX like AmpC (environmental/ HWW)	NG_068170.1	$bla_{\text{FOX-17}}$ (AmpC type β-lactamases)	98%	100%
E. coli-408_OXA-48 like (environmental/ RWS1)	NG_049539.1	$bla_{\text{OXA-244}}$ (carbapenemase_OXA-48 family class D β-lactamase)	99%	100%
E. coli-297_SHV-like (environmental/ WWTP)	NG_050087.1	$bla_{\text{SHV-5-like}}$ (ESBL)	97%	99.14%
E. coli-333_SHV-like (clinical/ wound)	NG_050008.1	$bla_{\text{SHV-13-like}}$ (ESBL)	97%	99.57%
E._coli-427_SHV-like_(environmental/HWW)	NG_050008.1	$bla_{\text{SHV-13-like}}$ (ESBL)	97%	99.13%
E. coli-739_SHV-like (environmetal/RWS1)	NG_050008.1	$bla_{\text{SHV-13}}$ (ESBL)	97%	100%
E. coli-203_TEM-like (clinical/ blood)	NG_050186.1	$bla_{\text{TEM-143-like}}$ (ESBL)	99%	99.88%
E. coli-494_TEM-like (environmental/ WWTP)	NG_050239.1	$Bla_{\text{TEM-207}}$ (ESBL)	99%	99.75%
E. coli-738_TEM-like (environmental/ RWS1)	NG_050186.1	$bla_{\text{TEM-143-like}}$ (ESBL)	99%	99.88%

Abbreviations: ESBL, extended-spectrum-β-lactamase; HWW, hospital wastewater; WWTP, wastewater treatment plant; RWS1, river water site 1; RWS2, river water site 2.

Supplementary Table S5a: Examination of the relationship between phylogenetic groups and origin of the sample; Sample and Group Crosstabulation

			Groups				Total
			**B1**	**B2**	**D**	**A**	
**Samples**	**RWS1**	Count	28	31	28	76	163
		Expected Count	23.1	47.3	24.7	67.9	163.0
		Residual	4.9	-16.3	3.3	8.1	
		Standardized Residual	1.0	-2.4	.7	1.0	
		**Adjusted Residual**	**1.3**	**-3.3**	**.8**	**1.5**	
	**RWS2**	Count	12	7	10	34	63
		Expected Count	8.9	18.3	9.5	26.2	63.0
		Residual	3.1	-11.3	.5	7.8	
		Standardized Residual	1.0	-2.6	.2	1.5	
		**Adjusted Residual**	**1.2**	**-3.3**	**.2**	**2.1**	
	**WWTP**	Count	25	38	30	78	171
		Expected Count	24.3	49.6	25.9	71.2	171.0
		Residual	.7	-11.6	4.1	6.8	
		Standardized Residual	.1	-1.6	.8	.8	
		**Adjusted Residual**	**.2**	**-2.3**	**1.0**	**1.2**	
	**HWW**	Count	20	26	5	54	105
		Expected Count	14.9	30.5	15.9	43.7	105.0
		Residual	5.1	-4.5	-10.9	10.3	
		Standardized Residual	1.3	-.8	-2.7	1.6	
		**Adjusted Residual**	**1.6**	**-1.1**	**-3.2**	**2.2**	
	**Clinical**	Count	6	84	24	25	139
		Expected Count	19.7	40.3	21.0	57.9	139.0
		Residual	-13.7	43.7	3.0	-32.9	
		Standardized Residual	-3.1	6.9	.6	-4.3	
		**Adjusted Residual**	**-3.8**	**9.2**	**.8**	**-6.4**	
**Total**		Count	91	186	97	267	641
		Expected Count	91.0	186.0	97.0	267.0	641.0

Supplementary Table S5b: Examination of the relationship between phylogenetic groups and origin of the sample; Pearson's chi-square test results

	Value	df	Asymptotic Significance (2-sided)
Pearson Chi-Square	110.630[a]	12	<.001
Likelihood Ratio	113.156	12	<.001
Linear-by-Linear Association	10.731	1	.001
N of Valid Cases	641		

[a] 0 cells (0.0%) have expected count less than 5. The minimum expected count is 8.94.

**Supplementary Table S6a.** Examination of the relationship between phylogenetic groups and resistance profile; Resistance profile and Group Crosstabulation							
			**Groups**				**Total**
			B1	A	B2	D	
**Environmental Resistance profile**	**MDR**	Count	6	53	23	20	102
		Expected Count	16.4	42.5	27.7	15.4	102.0
		Residual	-10.4	10.5	-4.7	4.6	
		Standardized Residual	-2.6	1.6	-.9	1.2	
		**Adjusted Residual**	**-3.1**	**2.3**	**-1.1**	**1.4**	
	**R**	Count	19	92	43	28	182
		Expected Count	29.2	75.8	49.4	27.5	182.0
		Residual	-10.2	16.2	-6.4	.5	
		Standardized Residual	-1.9	1.9	-.9	.1	
		**Adjusted Residual**	**-2.4**	**2.9**	**-1.3**	**.1**	
	**N-WT**	Count	1	8	3	1	13
		Expected Count	2.1	5.4	3.5	2.0	13.0
		Residual	-1.1	2.6	-.5	-1.0	
		Standardized Residual	-.8	1.1	-.3	-.7	
		**Adjusted Residual**	**-.8**	**1.5**	**-.3**	**-.8**	
	**WT**	Count	71	89	21	24	205
		Expected Count	32.9	85.4	55.6	31.0	205.0
		Residual	38.1	3.6	-34.6	-7.0	
		Standardized Residual	6.6	.4	-4.6	-1.3	
		**Adjusted Residual**	**8.8**	**.6**	**-6.6**	**-1.7**	
**Clinical Resistance profile**	**S clinical**	Count	1	11	38	6	56
		Expected Count	9.0	23.3	15.2	8.5	56.0
		Residual	-8.0	-12.3	22.8	-2.5	
		Standardized Residual	-2.7	-2.6	5.8	-.8	
		**Adjusted Residual**	**-3.0**	**-3.5**	**7.2**	**-1.0**	
	**MDR**	Count	0	2	11	6	19
		Expected Count	3.1	7.9	5.2	2.9	19.0
		Residual	-3.1	-5.9	5.8	3.1	

		Standardized Residual	-1.7	-2.1	2.6	1.8	
		**Adjusted Residual**	**-1.9**	**-2.8**	**3.1**	**2.0**	
	**R**	Count	5	12	35	12	64
		Expected Count	10.3	26.7	17.4	9.7	64.0
		Residual	-5.3	-14.7	17.6	2.3	
		Standardized Residual	-1.6	-2.8	4.2	.7	
		**Adjusted Residual**	**-1.9**	**-3.9**	**5.2**	**.9**	
**Total**		Count	103	267	174	97	641
		Expected Count	103.0	267.0	174.0	97.0	641.0

Supplementary Table S6b. Examination of the relationship between phylogenetic groups and resistance profile; Pearson's chi-square test results

	Value	df	Asymptotic Significance (2-sided)
Pearson Chi-Square	184.099[a]	18	<.001
Likelihood Ratio	179.391	18	<.001
Linear-by-Linear Association	4.029	1	.045
N of Valid Cases	641		

[a] 5 cells (17.9%) have expected count less than 5. The minimum expected count is 1.97.

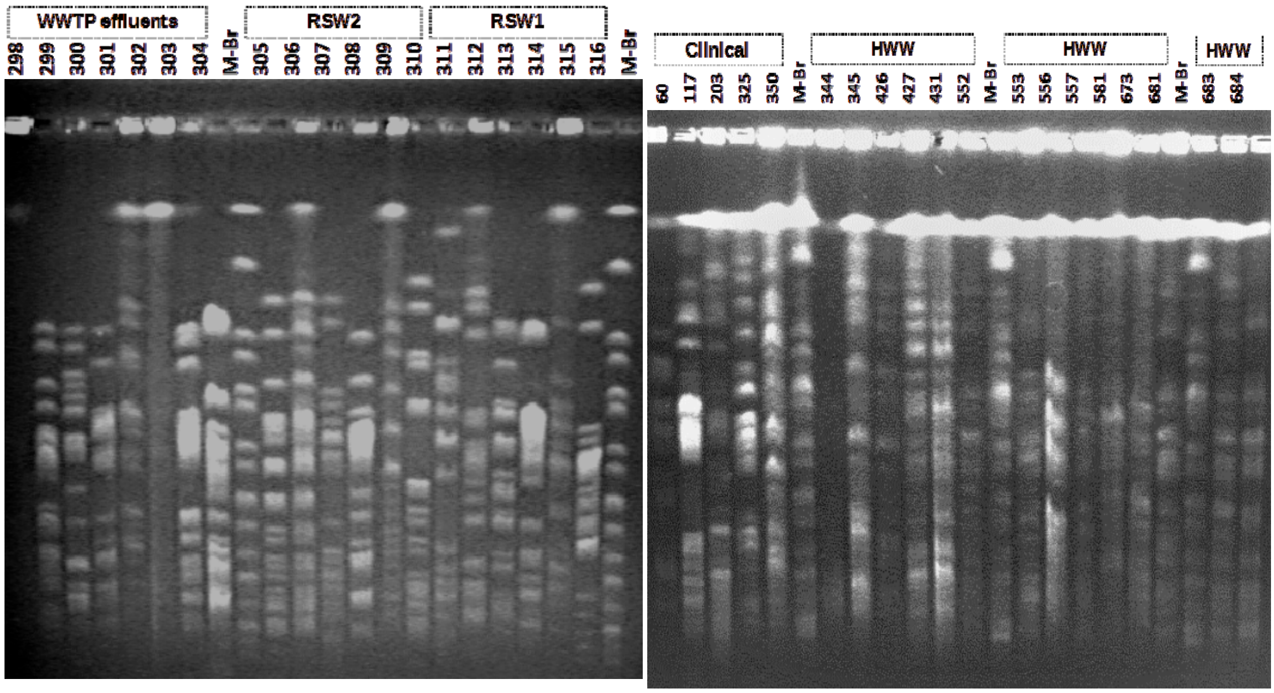

Supplementary Figure S1. PFGE analysis; Diverse PFGE patterns of *E. coli* isolated from clinical and environmental samples.

Abbreviations: HWW, hospital wastewater; WWTP, wastewater treatment plant; RWS1, river water site 1; RWS2, river water site 2.

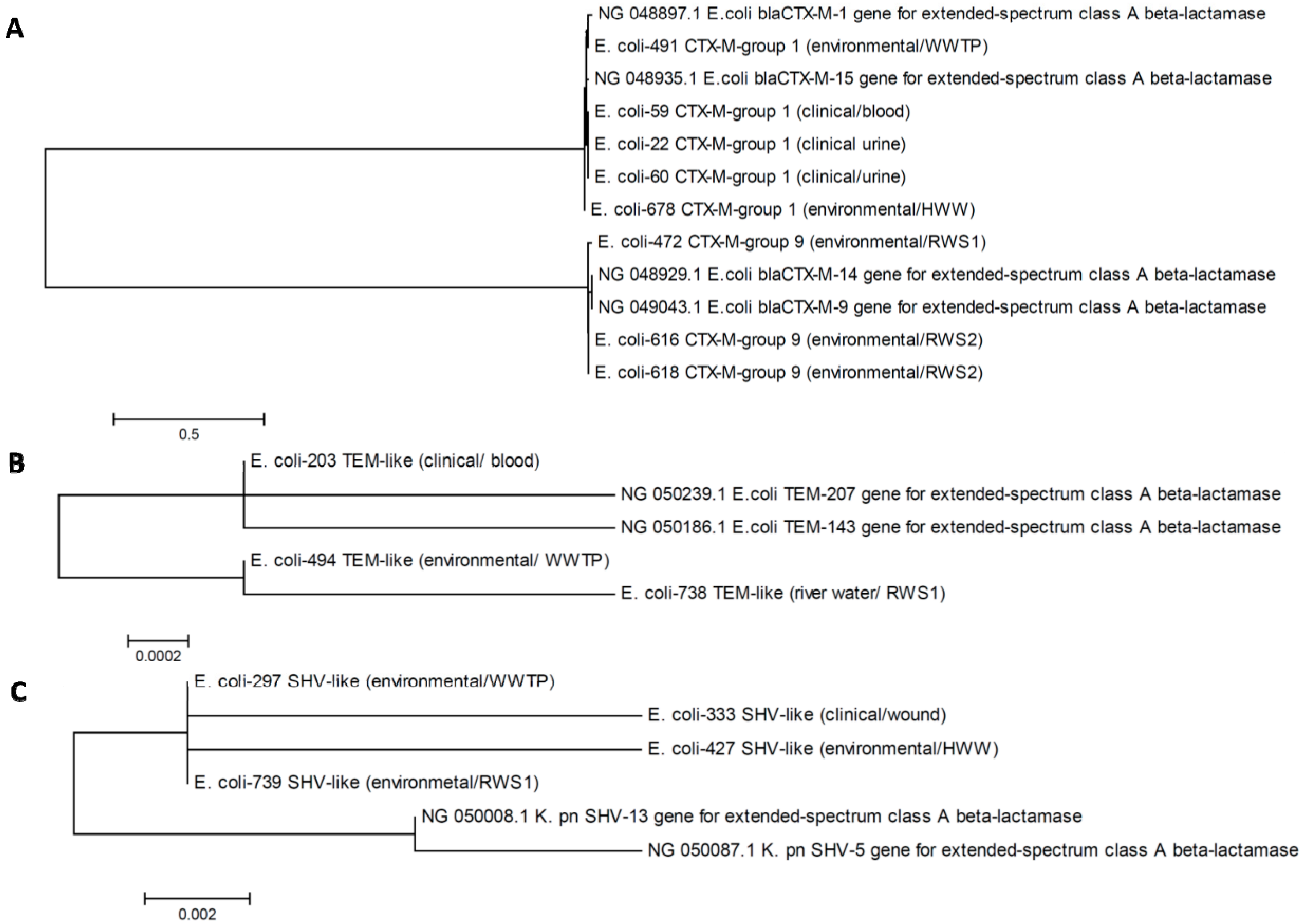

Supplementary Figure S2: Maximum Likelihood phylogenetic trees for A) *bla*$_{\text{CTX-M-groups}}$, B) *bla*$_{\text{TEM}}$ and C) *bla*$_{\text{SHV}}$ nucleotide sequences.
